# COVID-19 restrictions and age-specific mental health—U.S. probability-based panel evidence

**DOI:** 10.1038/s41398-021-01537-x

**Published:** 2021-08-04

**Authors:** Elvira Sojli, Wing Wah Tham, Richard Bryant, Michael McAleer

**Affiliations:** 1 UNSW Business School, University of New South Wales, Sydney, NSW Australia; 2 School of Psychology, University of New South Wales, Sydney, NSW Australia; 3 UNSW Traumatic Stress Clinic, Sydney, NSW Australia; 4 College of Management, Asia University, Taichung, Taiwan

**Keywords:** Scientific community, Depression

## Abstract

Social distancing, self-isolation, quarantining, and lockdowns arising from the COVID-19 pandemic have been common restrictions as governments have attempted to limit the rapid virus transmission. In this study, we identified drivers of adverse mental and behavioral health during the COVID-19 pandemic and whether factors such as social isolation and various restrictions serve as additional stressors for different age groups. Univariate and multivariate regression analyses were conducted on a unique dataset based on a national probability-based survey dedicated to understanding the impact of COVID-19 in the U.S., which includes 19 questions on the individual impact of restrictions, bans, and closures. The analysis used a moderate distress scale built on five questions related to mental health for 3,646 respondents. The mental health of young adults (18−34 years old) was the most affected by restrictions, while that of older adults (>55 years old) was less affected. In addition, demographic and health characteristics associated with differences in mental health varied by age group. The findings in this analysis highlight the differential mental health needs of different age groups and point to the marked necessity for differentiated and targeted responses to the mental health effects of COVID-19 by age group.

## Introduction

The COVID-19 public health crisis has forced governments the world over to impose social distancing, self-isolation, quarantining, and lockdowns in an attempt to limit the rapid virus transmission. While these restrictions were eased in July−August 2020, many such restrictions have been reintroduced in one form or another across countries since October 2020. Previous pandemics indicate that quarantine and social isolation lead to increased mental health problems; however, such prolonged and sweeping quarantine and lockdown measures have never been carried out [1].

**Fig. 1:**
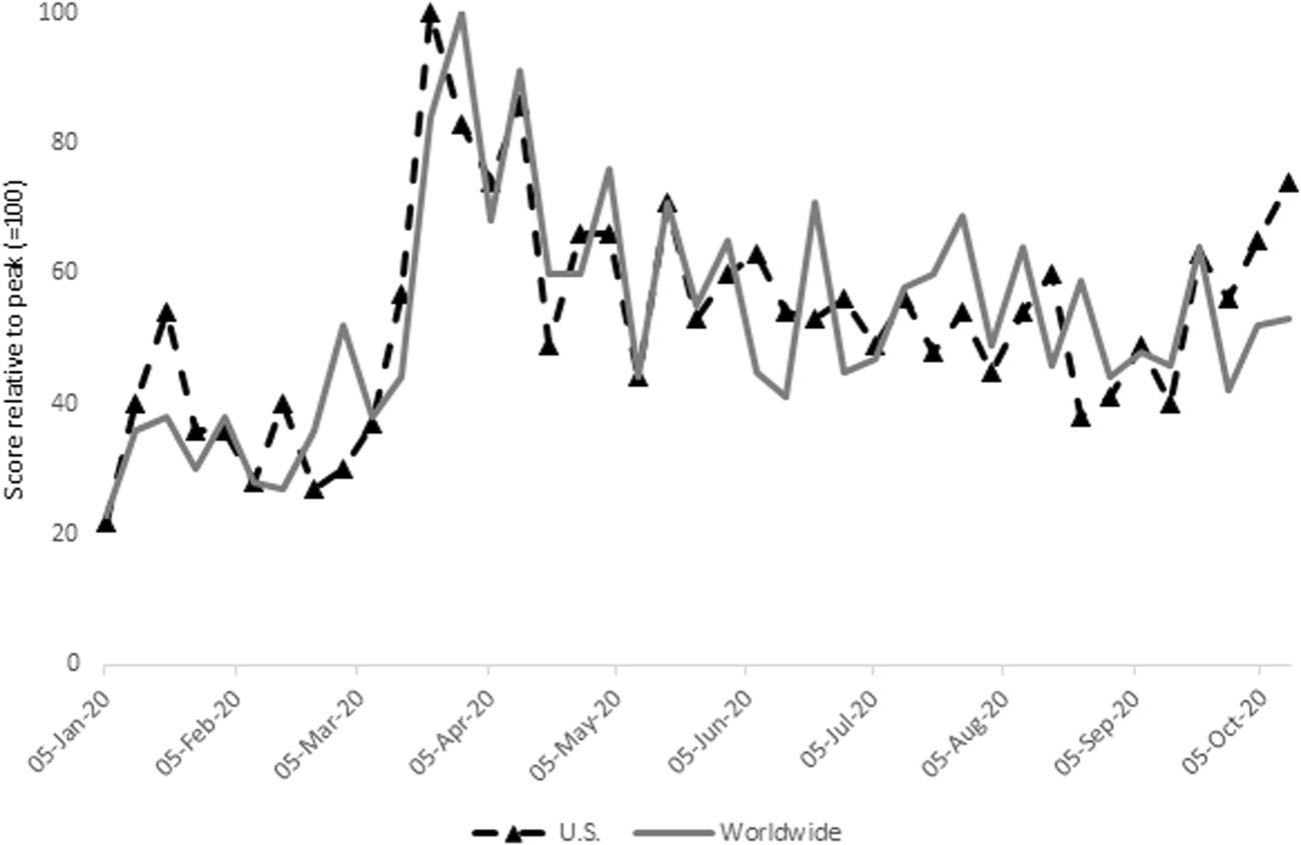
Trend for ‘mental health hotline’ Google searches. The figure shows the standardized weekly searches for the term ‘mental health hotline’ from January 05 to October 11, 2020, extracted from Google Trends. The Google Trends score represents search interest relative to the highest point on the chart for the given region and time period. A value of 100 is the peak popularity for the term. Worldwide (in solid gray line) presents the results for the global searches, and U.S. (dashed marked line) presents the results for the U.S. Source: Google Trends Accessed October 18, 2020.

Figure 1 shows that internet searches for the term ‘mental health hotline’ in Google Trends increased three-fold from February 29, 2020 to March 22, 2020, both globally and in the United States (U.S.). The Google Trend score represents search interest relative to the highest point on the chart for the given region and time period. While the peak search period was observed on March 22, 2020, the level of searches has remained elevated throughout the COVID-19 pandemic, and it started to pick up again in October 2020, when it was still higher than in the pre-COVID times. The figure provides some preliminary indications that the U.S. and the world population has and continues to experience increased mental health distress during the COVID-19 pandemic.

Indeed, systematic reviews demonstrate that approximately one in three people have experienced psychological distress during the pandemic [2]. Cohort studies indicate that the rates of common mental disorders (CMDs) have increased during the COVID-19 pandemic [3], with the greatest increases in those with no prior history of psychological problems [4]. These studies are based on surveys of mental health patterns during the COVID-19 pandemic, but most of these are community-based surveys of longitudinal cohorts that are susceptible to marked response bias [5–7]. There is the need to understand the effects of the COVID-19 pandemic on mental health in a representative population study, which is probability-based, as compared to community-based surveys that use weighting to be demographically representative. A recent study, using a representative population-based cohort survey of three waves between 18 March and 18 April 2020, showed the increase in incidences of acute stress and depressive symptoms during the COVID-19 pandemic in the U.S. [8]. Our study examined the effects of restriction and lockdowns arising from the COVID-19 pandemic on mental health across age cohorts, gender, and pre-existing comorbidities, including the self-reported history of prior mental health diagnosis, using a similar probability-based survey conducted over the period 20 April−8 June 2020. The main question investigated is whether the mental health of all adults affected by the COVID-19 restrictions was impacted in the same way. On the basis that younger people are at greater risk of mental health problems during the pandemic [6], we expected the effects of social restrictions to be greater on younger people.

A large literature investigates the psychological effects of large-scale health shocks (SARS, MERS) or disasters (natural and non), especially post-traumatic stress disorders (PTSD) [9–11]. Very few studies in the literature investigate the age effects separately, and the relation between age and mental health outcomes in these studies is ambiguous. Our work focuses on the age-related effects of the COVID-19 pandemic, adding to this literature evidence of age-group differences in CMDs.

## Materials and methods

### Survey details

The COVID-19 Household Impact Survey is an effort to provide national and regional statistics about physical health, mental health, economic security, and social dynamics in the U.S., as described in Wozniak et al. [12–14]. The survey is funded by the Data Foundation and conducted by the National Opinion Research Center (NORC) at the University of Chicago. The survey targets a nationally representative sample of adults aged ≥ 18 in the U.S. The sample is selected from the AmeriSpeak Panel using sampling strata based on age, race/ethnicity, education, and gender (with 48 sampling strata in total). The sample coverage is about 97% of the U.S. household population, and the recruitment rate is 21.5% across 30,076 individuals.

The analysis is based on three waves of the survey conducted during the periods: 20−26 April, 4−10 May, and 30 May−8 June 2020, with 6,475 respondents in total. The samples are drawn independently for each week to provide independent, cross-sectional estimates for each wave. Participants provided informed consent when they joined the NORC panel and were informed that their identities would remain confidential. All research activities were reviewed and approved by the NORC Institutional Review Board for Human Subjects research. The data is available from the NORC website: https://www.norc.org/Research/Projects/Pages/covid-impact-survey.aspx, and details of each survey wave are available under the Resources tab: COVID Impact Survey Week 1 Data and Methods, COVID Impact Survey Week 2 Data and Methods, COVID Impact Survey Week 3 Data and Methods.

### Measures

#### Mental distress

The survey asked respondents to consider: “In the past 7 days, how often have you:

(1) Felt nervous, anxious, or on edge;

(2) Felt depressed;

(3) Felt lonely;

(4) Felt hopeless about the future;

(5) Had physical reactions such as sweating, trouble breathing, nausea or a pounding heart when thinking about your experience with the coronavirus pandemic”.

The response options were: (1) Not at all or less than 1 day; (2) 1−2 days; (3) 3−4 days; (4) 5−7 days, which have been codified as 0, 1, 2, and 3, respectively. The responses to the five symptom questions were used to create a Psychological Distress Scale called T5, which is a five-item scale that is scored on a four-point scale (total range: 0−15). Higher scores indicate higher mental distress. The summary statistics are presented in Panel A of Supplementary Table S1.

The mental health constructed variables are highly correlated with each other, with correlations varying between 0.37 (*p*-val. < 0.01) and 0.62 (*p*-val. < 0.01), in Panel B of Supplementary Table S1. They are also highly correlated with the composite score T5, with correlations varying between 0.57 (*p*-val. < 0.01) and 0.82 (*p*-val. < 0.01).The Cronbach alpha results demonstrate excellent internal consistency and reliability, as the raw and standardized alpha scores are around 0.84 in panel C of Supplementary Table S1. All the variables are equally important for the internal consistency of a composite measure in panel D of Supplementary Table S1.

The computed T5 composite score was compared against a criterion measure based on self-reported previous mental health diagnosis. We used question PHYS8H:

‘Has a doctor or other health care provider ever told you, you have a mental health condition?’ Respondents that answered “Yes” were categorized as having a self-reported history of receiving a mental health condition diagnosis, and this was our criterion measure. The receiver operating characteristic (ROC) curve analysis was used to validate the T5 composite score against the criterion measure. Youden’s *J* statistic indicated that the optimum threshold was when the T5 score was ≥3 (sensitivity = 0.70, specificity = 0.73, total classification rate = 0.70, AUC = 0.77, and Cronbach’s alpha = 0.84). Thus, moderate distress was defined as an indicator variable equal to one when the T5 score was ≥3, and zero otherwise.

### Explanatory variables

Apart from the demographic (gender and race) and household composition (alone, with other adults, with 1−2, or more children) information provided by the survey respondents, respondents were also asked the following 19 questions about the closures and restrictions of various facilities and public services:

### In the past 7 days, have your personal plans been changed or affected by the following types of restrictions, or not?

(1) K-12 school closure, (2) Pre-K or childcare closure, (3) College or training closure, (4) Ban on gatherings of 250 people or more, (5) Ban on gatherings of 50 people or more, (6) Ban on gatherings of 10 people or more, (7) Closure of place of worship, (8) Reduced public transportation, (9) Other reduced public services, (10) Closure of bars, (11) Closure of restaurants, (12) Closure of gyms or fitness facilities, (13) Closure of other businesses, (14) Cancelled sports events, (15) Closure of work, (16) Work from home requirements, (17) Quarantine requirements or stay-at-home orders, (18) International travel restrictions or bans, and (19) Domestic travel restrictions or bans. For each question, we examine individuals who were affected by the closure relative to those who were not affected (using the answer “NO” as a benchmark).

In addition, respondents were asked about their current employment, likely future job prospects, and the state of their physical health through the following questions: “In the past 7 days, did you do any work for pay at a job or business?” (response options: “Yes, I worked for someone else for wages, salary, …,” “Yes, I worked as self-employed in my own business, …,” “No, I did not work for pay last week.” The latter was used as the benchmark.), “Think about 3 months from now, how likely do you think it is that you will be employed at that time?” (response options: “Extremely likely,” “Very likely,” “Moderately likely,” “Not too likely,” “Not likely at all”. The latter was used as the benchmark.), and **“**Would you say your health, in general, is excellent, very good, good, fair, or poor?**”** (poor physical health was used as the benchmark).

### Statistical analysis

The differential mental distress response across age groups was evaluated through multivariate logistic regressions, with the dependent variable being the moderate mental distress indicator. The analysis was carried out using SAS 9.4. The independent variables were the COVID-19 restriction variables. Potential confounding effects were controlled for through covariates for gender, race, household composition, prior mental health diagnoses, physical health, employment in the past 7 days, and job prospects in 90 days. We also controlled for the survey wave period using survey wave fixed effects, where the first survey in April 2020 was the benchmark. Regressions were estimated separately for each age group.

Throughout the survey, respondents had the response options “Not sure” and “Skipped on the web” available to them. The regression analysis focused on the responses “Yes” and “No,” i.e., deleting the not sure and skipped responses. This is one way of handling “Do not know” type responses in a regression setting [15, 16]. These responses can be excluded as missing data, presumed to mean “neither” and recoded as a neutral midpoint in a Likert response scale, or treated as a meaningful categorical response. However, there is no clear best practice, and each approach has limitations. Most commonly, “Do not know” responses are treated as missing data and excluded from the analysis [17], even though the best approach would be to prompt further answers to explain the “Do not know” response [18]. Exclusion of the “Not sure” and “Skipped on the web” responses from the analyses reduces statistical power and could increase the chance of making a Type 2 error (i.e., failure to detect an effect that is present), but it should not affect inference on the found/identified effects [19]. The final regression analysis included 3,646 respondents. Robustness analysis was conducted using the full sample of responses and treating “Not sure” and “Skipped on the web” as a meaningful categorical response, with qualitatively similar results.

## Results

The prevalence of moderate mental distress during the COVID-19 lockdowns for U.S. adults across different age groups for the full sample of 6,475 respondents is presented in Table 1. Approximately 37% of the sample reported moderate mental distress. The mean T5 score for the population was 2.65 (95% CI 2.57−2.73). The 18−24-year-old group was the most heavily affected, with more than one-half (52.42%) feeling moderate mental distress. The prevalence decreased consistently across the age cohorts, with less than 25% of the >65 years old feeling mentally distressed.

**Table 1:** Moderate mental distress prevalence.

	Prevalence of mental distress			T5 composite score	
	*N*	Estimate (%)	Confidence interval	Mean	Confidence interval
Full sample	6,475	36.79	[95%, 35.12−38.46]	2.65	[95%, 2.57−2.73]
Age in years					
18−24	362	52.42	[95%, 44.92−59.91]	4.08	[95%, 3.69−4.47]
25–34	1437	48.28	[95%, 44.73−51.84]	3.48	[95%, 3.29−3.67]
35–44	1066	42.85	[95%, 38.80−46.90]	3.15	[95%, 2.93−3.37]
45−54	951	37.02	[95%, 32.92−41.12]	2.63	[95%, 2.41−2.85]
55−64	1245	25.95	[95%, 22.67−29.22]	2.04	[95%, 1.88−2.20]
65−74	948	22.09	[95%, 18.50−25.67]	1.67	[95%, 1.51−1.83]
75+	466	23.60	[95%, 18.29−28.91]	1.37	[95%, 1.18−1.56]
Broader age groups					
18−34	1799	49.84	[95%, 46.27−53.42]	3.60	[95%, 3.43−3.78]
35–54	2017	39.96	[95%, 37.07−42.85]	2.91	[95%, 2.75−3.06]
>55	2659	24.08	[95%, 21.88−26.28]	1.79	[95%, 1.69−1.90]

The table presents the prevalence of moderate mental distress and the T5 composite score for the full sample and across age groups. *N* denotes the number of observations.

To analyze the differential relations of the lockdown measures for each age category, the sample was separated into three broad age groups: 18−34 (young adults), 35−54 (middle-age adults), and >55 (older adults) years old [16]. The moderate mental distress prevalence rates across these groups were 50% (95% CI 46.27−53.42), 40% (95% CI 37.07−42.85), and 24% (95% CI 21.88−26.28), respectively. The T5 composite scores were 3.60 (95% CI 3.43−3.78), 2.91 (95% CI 2.75−3.06), and 1.79 (95% CI 1.69−1.90) for 18−34, 35−54, and >55 years old, respectively. It is important to note that the different age groups had non-overlapping 95% CIs for prevalence as well as mean scores for mental distress, indicating the need to analyze the groups separately. Supplementary Table S2 shows that the sample and age subgroup mental distress characteristics remain qualitatively similar for the analytical sample used in the regression analysis, as described in the Statistical analysis section.

The coefficient estimates from the multivariate logistic regressions of the moderate mental distress measure on COVID-19 restrictions for each age group are presented in Table 2. The largest association was found between moderate mental health symptoms and a self-reported history of having received a mental health condition diagnosis. The estimated association varied between a four- to six-fold increase in the probability of mental distress, when having a self-reported history of receiving a mental health condition diagnosis in comparison to not having such a self-reported history. This result was not surprising given the validity check in the prior section.

**Table 2:** COVID-19 Restrictions and mental health across age groups.

		18–34				35–54				>55			
		Coeff.	S.E.	*p*-val.	Odds-ratio	Coeff.	S.E.	*p*-val.	Odds-ratio	Coeff.	S.E.	*p*-val.	Odds-ratio
	Intercept	−0.58	0.97	0.55		**−1.06**	**0.61**	**0.08**		**−1.60**	**0.89**	**0.07**	
Closures	K-12 school closure	0.18	0.16	0.27	1.20	−0.16	0.16	0.33	0.85	−0.08	0.16	0.59	0.92
	Pre-K or child care closure	−0.01	0.17	0.97	0.99	0.21	0.16	0.18	1.24	−0.02	0.16	0.92	0.98
	College or training closure	−0.13	0.17	0.45	0.88	0.14	0.16	0.40	1.15	0.09	0.16	0.59	1.09
	Ban on gatherings of >250 people	0.04	0.17	0.80	1.04	0.27	0.16	0.10	1.31	**0.30**	**0.16**	**0.06**	**1.35**
	Ban on gatherings of >50 people	−0.10	0.17	0.55	0.90	−0.02	0.16	0.91	0.98	0.09	0.16	0.55	1.10
	Ban on gatherings of >10 people	−0.01	0.17	0.93	0.99	−0.18	0.16	0.26	0.84	−0.07	0.16	0.66	0.93
	Closure of place of worship	−0.21	0.17	0.22	0.81	**0.28**	**0.16**	**0.08**	**1.32**	0.25	0.16	0.11	1.29
	Reduced public transportation	**0.42**	**0.17**	**0.01**	**1.53**	**−0.31**	**0.16**	**0.06**	**0.74**	0.17	0.16	0.28	1.19
	Other reduced public services	**0.38**	**0.17**	**0.03**	**1.46**	**0.30**	**0.16**	**0.06**	**1.35**	0.05	0.16	0.78	1.05
	Closure of bars	−0.19	0.17	0.28	0.83	0.08	0.16	0.63	1.08	−0.08	0.16	0.64	0.93
	Closure of restaurants	**0.40**	**0.17**	**0.02**	**1.50**	0.12	0.16	0.45	1.13	0.04	0.16	0.80	1.04
	Closure of gyms/fitness facilities	0.15	0.17	0.37	1.16	−0.16	0.16	0.32	0.85	−0.03	0.16	0.87	0.97
	Closure of other businesses	−0.07	0.17	0.70	0.94	**0.30**	**0.16**	**0.06**	**1.35**	−0.11	0.16	0.49	0.90
	Canceled sport events	0.18	0.17	0.29	1.20	0.06	0.16	0.70	1.07	0.05	0.16	0.75	1.05
	Closure of work	0.00	0.17	0.99	1.00	−0.02	0.16	0.89	0.98	**0.28**	**0.16**	**0.08**	**1.33**
	Work from home requirements	**−0.38**	**0.17**	**0.03**	**0.68**	0.15	0.15	0.32	1.17	0.14	0.16	0.36	1.15
	Quarantine/stay-at-home orders	−0.14	0.17	0.40	0.87	0.11	0.16	0.49	1.12	0.09	0.16	0.58	1.09
	International travel restrictions	**0.41**	**0.18**	**0.02**	**1.50**	**0.35**	**0.17**	**0.04**	**1.42**	0.10	0.16	0.53	1.11
	Domestic travel restrictions	0.08	0.18	0.67	1.08	−0.09	0.18	0.63	0.92	0.27	0.17	0.11	1.31
Job prospect 90 days	Extremely likely	−0.10	0.32	0.77	0.91	0.32	0.29	0.27	1.38	−0.10	0.27	0.72	0.91
	Very likely	0.42	0.33	0.21	1.52	0.33	0.30	0.28	1.39	**0.52**	**0.27**	**0.05**	**1.68**
	Moderately likely	0.54	0.34	0.11	1.71	**0.88**	**0.30**	**0.00**	**2.40**	**0.45**	**0.24**	**0.07**	**1.56**
	Not too likely	0.01	0.41	0.99	1.01	0.58	0.35	0.10	1.78	0.18	0.27	0.49	1.20
Gender	Male	−0.08	0.08	0.28	0.85	**−0.17**	**0.07**	**0.01**	**0.71**	**−0.25**	**0.07**	**0.00**	**0.61**
Race	Non-Hispanic white	**0.82**	**0.38**	**0.03**	**2.27**	0.43	0.38	0.26	1.54	0.76	0.79	0.34	2.13
	Non-Hispanic black	0.46	0.42	0.28	1.59	−0.29	0.42	0.48	0.75	0.22	0.81	0.79	1.25
	Hispanic	0.80	0.40	0.05	2.22	0.12	0.41	0.77	1.13	0.33	0.82	0.68	1.40
Mental health	Prior mental health diagnose	**1.53**	**0.20**	**0.00**	**4.60**	**1.43**	**0.18**	**0.00**	**4.17**	**1.75**	**0.21**	**0.00**	**5.78**
Physical health	Excellent	**−1.88**	**0.87**	**0.03**	**0.15**	**−1.63**	**0.50**	**0.00**	**0.20**	**−1.72**	**0.39**	**0.00**	**0.18**
	Very good	**−1.44**	**0.86**	**0.09**	**0.24**	**−1.07**	**0.46**	**0.02**	**0.34**	**−1.44**	**0.33**	**0.00**	0.24
	Good	−1.16	0.86	0.18	0.32	−0.43	0.46	0.35	0.65	**−0.98**	**0.33**	**0.00**	**0.37**
	Fair	−0.80	0.88	0.36	0.45	−0.32	0.48	0.50	0.73	−0.33	0.34	0.34	0.72
Employemnent	Worked for someone else	0.11	0.18	0.55	1.12	−0.14	0.18	0.43	0.87	−0.12	0.24	0.62	0.89
	Worked as self-employed	−0.13	0.31	0.69	0.88	−0.37	0.27	0.17	0.69	−0.05	0.31	0.86	0.95
House-hold	Alone	**0.46**	**0.20**	**0.02**	**1.58**	0.11	0.18	0.54	1.12	0.14	0.26	0.61	1.15
	+1 other adult only	0.21	0.20	0.29	1.24	−0.12	0.19	0.52	0.89	−0.03	0.27	0.90	0.97
	1 or 2 kids	−0.14	0.23	0.56	0.87	−0.02	0.20	0.91	0.98	0.33	0.60	0.58	1.39
Fixed effect	8-Jun-20	−0.03	0.11	0.79	0.85	−0.01	0.10	0.96	0.95	0.07	0.10	0.45	1.01
	10-May-20	−0.10	0.10	0.31	0.79	−0.04	0.09	0.68	0.92	−0.14	0.10	0.14	0.81

The table presents the effects of COVID-19 restrictions on moderate mental distress obtained from a cross-sectional logistic regression of the moderate mental distress dummy on COVID-19 restrictions and control variables. The regression output includes: coefficient estimates (Coeff.), standard errors (S.E.), probability of the coefficient is different from zero (*p*.-val.), and the odds-ratios. The estimated coefficients are obtained by age group for 18−34, 35−54, and >55 years old separately through three separate regressions. Coefficients in bold are significantly different from zero at the 10% significance level.

Better physical health was also highly correlated with lower levels of moderate mental distress. Excellent physical health was strongly correlated with a lower probability of moderate mental distress, in comparison with poor physical health (odds ratio from 0.15−0.20), i.e., an individual with poor physical health was five times more likely to feel moderate mental distress. Very good physical health in comparison with poor physical health was related to a lower likelihood of moderate mental distress for all age groups. For older adults, good physical health was related to a lower likelihood of mental distress.

Non-Hispanic white young adult respondents had a higher probability of moderate mental distress, as compared with young adult Asian respondents (coeff. = 0.82, *p*-val. = 0.03, odds-ratio = 2.27, 95% CI 1.07−4.98). Middle-age (coeff. = −0.17, *p*-val. = 0.01, odds-ratio = 0.71, 95% CI 0.54−0.93) (coeff. = −0.17, *p*-val. = 0.01, odds-ratio = 0.71, 95% CI 0.54−0.93) and older male (coeff. = −0.25, p-val. = <0.001, odds-ratio = 0.61, 95% CI 0.47−0.79) (coeff. = −0.25, *p*-val. = <0.001, odds-ratio = 0.61, 95% CI 0.47−0.79) respondents tended to have lower rates of moderate mental distress than did female respondents. There was no gender effect for young adults.

Job certainty in the next 90 days was related to the mental distress of the three age groups differently, after controlling for already being employed or not. The mental health of young adults was not related to the expected likelihood of obtaining a job in the next 90 days. On the contrary, among middle-aged adults, those who were moderately likely to have a job were more likely (coeff. = 0.88, *p*-val. = <0.001, odds-ratio = 2.40, 95% CI 1.33−4.35) to experience moderate mental distress than those who were not likely at all to have a job in 90 days. Older adults who were very likely (coeff. = 0.52, *p*-val. = 0.05, odds-ratio = 1.68) and moderately likely (coeff. = 0.45, *p*-val. = 0.07, odds-ratio = 1.56) to have a job in 90 days were more likely to feel moderate mental distress than the benchmark group.

**Fig. 2:**
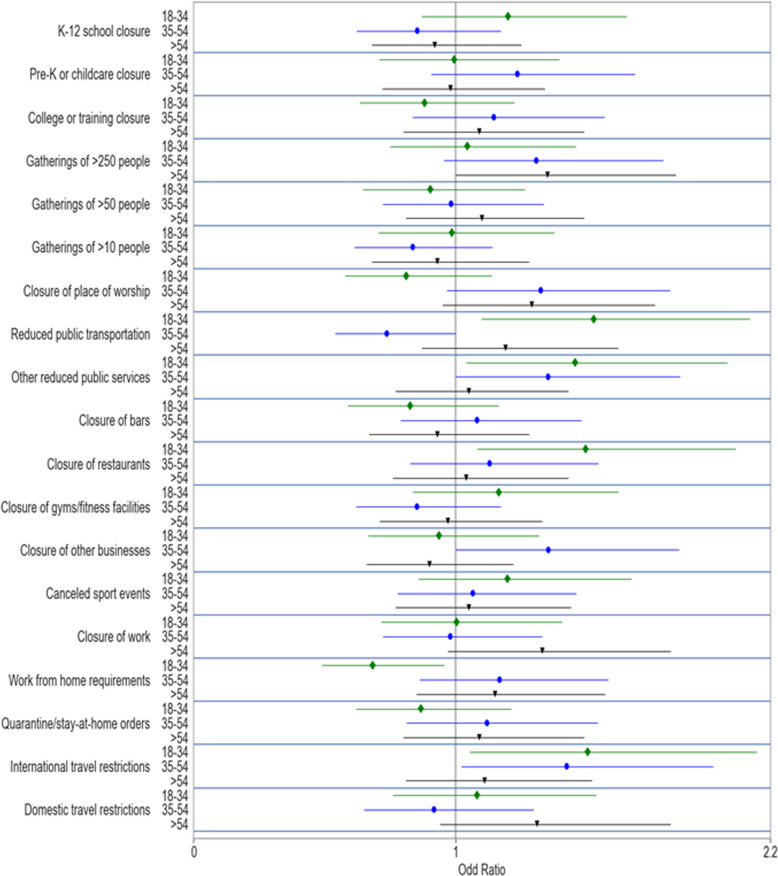
Odds-ratio of COVID-19 restrictions effect on mental health by age group. The figure shows the odds-ratio (and 95% confidence interval) for the effects of COVID-19 restrictions effect on moderate mental distress obtained from a cross-sectional logistic regression of the moderate mental distress variable on COVID-19 restrictions and control variables. The estimated coefficients are obtained by age group for 18−34, 35−54, and >55 years old separately through three separate regressions. Table 2 presents all the regression coefficients.

The odds-ratios and confidence intervals from the multivariate logistic regression estimates (in Table 2) of the moderate mental distress measure on COVID-19 restrictions for each age group are presented in Fig. 2. The COVID-19 restrictions affected the different age groups differently. The mental health of older adults was significantly correlated to two restrictions. Only the ban on gatherings of more than 50 people (coeff. = 0.30, p-val. = 0.06, odds-ratio = 1.35) and closure of work (coeff. = 0.28, *p*-val. = 0.08, odds-ratio = 1.33) were related to the mental distress of this group. Moderate mental distress for middle-aged adults was strongly related to international travel restrictions, whose closure increased their moderate mental distress (coeff. = 0.35, *p*-val. = 0.04, odds-ratio = 1.42). Their mental distress was also related to the closure of other businesses (coeff. = 0.30, *p*-val. = 0.06, odds-ratio = 1.35), closure of places of worship (coeff.=0.28, *p*-val. = 0.08, odds-ratio = 1.32), and the reduction in public services (coeff. = 0.30, *p*-val. = 0.06, odds-ratio = 1.35) other than public transport in equal measure. The reduction in public transport was related to a lower likelihood of moderate mental distress (coeff. = −0.31, *p*-val. = 0.06, odds-ratio = 0.74). The closure of bars, restaurants, gyms, and even schools and childcare, was not related to the mental wellness of this age group.

Finally, the mental health distress of young adults was related to an almost completely different set of restrictions. Their mental distress was negatively related to the most, and a wide array of, restrictions. Reductions in public transport (coeff. = 0.42, *p*-val. = 0.01, odds-ratio = 1.53) and in other public services (coeff. = 0.38, *p*-val. = 0.03, odds-ratio = 1.46) increased their likelihood of exhibiting moderate mental distress. The closure of restaurants was also related to an increase in the likelihood of young adults to suffer from moderate mental distress (coeff. = 0.40, *p*-val. = 0.02, odds-ratio = 1.50), as did international travel restrictions (coeff. = 0.41, *p*-val. = 0.02, odds-ratio = 1.50). Finally, work from home requirements were related to lower moderate mental distress (coeff. = −0.38, *p*-val. = 0.03, odds-ratio = 0.68).

## Discussion and limitations

The findings in this analysis highlight the differential impact of COVID-19 restrictions on the mental health of adults of different age groups. We find marked differences in how COVID-19 restrictions and lockdowns were related to mental health outcomes across different segments of the population. Moderate mental distress due to COVID-19 lockdowns, appears to be highest in individuals with a self-reported history of receiving a mental health condition diagnosis, while better physical health is related to lower likelihoods of moderate mental distress. The restrictions were correlated the most with the mental health distress of young adults and the least with the mental health distress of older adults. This may be attributed to younger people relying more on mobility for social interaction, travel, and leisure activities, and the loss of these activities adversely impacts on mental health. The demographic and health characteristics that matter for each age group also differ. These findings provide important representative data that supports projected impacts of social isolation and restrictions arising from the COVID-19 pandemic and provide evidence for shaping public healthcare policy.

The results should be taken with caution for several reasons. First, a diagnostic evaluation for an anxiety disorder or depressive disorder was not conducted directly. However, clinically validated screening instruments were used to assess five symptoms. Second, while the study controls for many factors that affect mental disorders, there may be factors related to the COVID-19 pandemic that have not been included in the analysis, which may confound the analysis. Third, we do not have information on the differences across these age groups prior to the pandemic, thus time-series comparisons to pre-pandemic times cannot be drawn. Fourth, the average response rate of surveyed participants was 20%. We use panel-based sampling weights in our analysis; however, issues related to response selection bias may remain.

The findings complement prior work [6, 8, 20] with a detailed analysis of COVID-19 restriction-specific conditioning factors that are related to the mental status of adults in different age groups. These findings underscore the need to focus on preventative actions to reduce the mental health burden during the pandemic. For example, the data indicate that specific attention needs to be given to the mental health of people (especially older ones) experiencing poor physical health, the specific impacts of social restrictions on younger people, and the potential adverse impacts of job stress on younger adults during the pandemic. Age-specific health policies and interventions may be optimal rather than universal programs that presume they are equally applicable to the health needs of all age groups.

## Supplementary information

Supplementary materials

## Data Availability

The data is available at the NORC website: https://www.norc.org/Research/Projects/Pages/covid-impactsurvey.aspx.

## Code availability

The code for the results presented is available at Dr. Sojli’s website https://sites.google.com/site/esojli/publications.
