# Supplementary material for: COVID-19 restrictions and age-specific mental health—U.S. probability-based panel evidence
Source: Transl Psychiatry. 2021 Aug 4;11:418. doi: 10.1038/s41398-021-01537-x (PMC8336527; doi:10.1038/s41398-021-01537-x)
Supplement: Supplementary file 1 — Supplementary materials [file 41398_2021_1537_MOESM1_ESM.pdf]

Supplementary Table S1. Summary statistics of mental health variables

Panel A. Variable Characteristics

In the past 7 days, how often have you?	Mean	Std. Dev.
Felt nervous, anxious, or on edge	0.62	0.91
Felt depressed	0.62	0.91
Felt lonely	0.62	0.92
Felt hopeless about the future	0.63	0.92
Had physical reactions such as sweating, trouble breathing, nausea or a pounding heart when thinking about your experience with the coronavirus pandemic	0.16	0.51
Mental health composite score, T5	2.65	3.23

Panel B. Correlations

	Composite	Nervous	Depressed	Lonely	Hopeless
Nervous	0.82***				
Depressed	0.82***	0.60***			
Lonely	0.83***	0.59***	0.59***		
Hopeless	0.83***	0.59***	0.59***	0.62***	
Physical reaction	0.57***	0.37***	0.37***	0.37***	0.38***

Panel C. Cronbach's Coefficient Alpha

Standardized	0.84	Raw	0.84

Panel D. Cronbach's Coefficient Alpha with Deleted Variable

Deleted Variable	Raw	Standardized
Nervous	0.79	0.79
Depressed	0.79	0.79
Lonely	0.79	0.79
Hopeless	0.78	0.79
Physical reaction	0.85	0.85

*** denote statistically different from zero with p-value ≤ 0.01.

Supplementary Table S2. Moderate Mental Distress Prevalence for the Analytical Sample

The table presents the prevalence of moderate mental distress and the T5 composite score for the analytical sample as described in Section Statistical analysis and across age subgroups in this sample. N denotes the number of observations.

	Prevalence of mental distress			T5 Composite Score	
	N	**Estimate (%)**	**Confidence Interval**	**Mean**	**Confidence Interval**
Analytical sample	3,646	34.25	[95%, 33.23-35.27]	2.47	[95%, 2.36-2.57]
Age in years					
18-24	155	50.86	[95%, 45.96-56.02]	4.04	[95%, 3.47-4.60]
25–34	815	44.75	[95%, 42.57-46.93]	3.21	[95%, 2.97-3.44]
35–44	659	41.07	[95%, 38.64-43.50]	2.91	[95%, 2.65-3.19]
45-54	529	34.24	[95%, 31.68-36.79]	2.52	[95%, 2.24-2.81]
55-64	647	25.26	[95%, 23.09-27.44]	1.95	[95%, 1.74-2.16]
65-74	578	20.09	[95%, 17.99-22.19]	1.53	[95%, 1.33-1.73]
75+	263	21.52	[95%, 18.17-24.87]	1.26	[95%, 1.02-1.51]
Broader age groups					
18-34	970	46.66	[95%, 44.47-48.85]	3.34	[95%, 3.12-3.56]
35–54	1,188	37.71	[95%, 35.94-39.48]	2.74	[95%, 2.54-2.94]
>55	1,488	22.52	[95%, 21.13-23.90]	1.67	[95%, 1.54-1.80]
